# Overcoming immune evasion with innovative multi-target approaches for glioblastoma

**DOI:** 10.3389/fimmu.2025.1541467

**Published:** 2025-01-22

**Authors:** Hai Su, Yin Peng, Yilong Wu, Xiaoli Zeng

**Affiliations:** ^1^ Department of Neurosurgery, Yongchuan Hospital of Chongqing Medical University, Chongqing, China; ^2^ Department of Oncology, The First Affiliated Hospital of Gannan Medical University, Ganzhou, Jiangxi, China; ^3^ Jiangxi “Flagship” Oncology Department of Synergy for Chinese and Western Medicine, The First Affiliated Hospital of Gannan Medical University, Ganzhou, Jiangxi, China; ^4^ Department of Oncology, Jiangxi Clinical Research Center for Cancer, The First Affiliated Hospital of Gannan Medical University, Ganzhou, Jiangxi, China

**Keywords:** glioblastoma, immune evasion, immunotherapy, PD-1, immune checkpoint

## Abstract

Glioblastoma (GBM) cells leverage complex endogenous and environmental regulatory mechanisms to drive proliferation, invasion, and metastasis. Tumor immune evasion, facilitated by a multifactorial network, poses a significant challenge to effective therapy, as evidenced by the limited clinical benefits of monotherapies, highlighting the adaptive nature of immune evasion. This review explores glioblastoma’s immune evasion mechanisms, the role of ICIs in the tumor microenvironment, and recent clinical advancements, offering theoretical insights and directions for monotherapy and combination therapy in glioblastoma management.

## 1 Introduction

Glioblastoma, the most common primary tumor of the central nervous system (CNS), originate from glial cells and account for over 80% of brain tumors. According to the World Health Organization (WHO), gliomas are categorized into four grades, with grade IV, known as glioblastoma, comprising 60–70% of all gliomas. GBM, the most prevalent primary malignant CNS tumor, manifests with symptoms such as progressive neurological deficits, headaches, nausea, vomiting, cognitive impairment, and seizures, depending on tumor stage and location. The incidence of GBM is approximately 3.21 cases per 100,000 individuals, with prevalence increasing with age and a median diagnosis age of 65 years. Males are more commonly affected than females, with a male-to-female ratio of 1.28:1 (1).

Despite advancements in surgical resection, radiotherapy, and temozolomide (TMZ)-based chemotherapy, GBM prognosis remains poor, with a median survival of 15 months and a five-year survival rate of 9.8% (2, 3). Challenges include its high migratory and invasive properties, preventing complete resection, and frequent localized recurrence near resected tissue, leading to patient mortality (4). Anti-angiogenic agents like bevacizumab and tumor-treating fields (TTF) have shown limited success in prolonging survival, emphasizing the need for innovative therapies (5, 6). Immunotherapy, a rapidly advancing field, aims to enhance immune responses to suppress tumor progression (7–12). However, GBM’s unique immune evasion mechanisms present significant obstacles. Immunotherapeutic approaches, including immune checkpoint inhibitors (ICIs), cytokine-based therapies, dendritic cell vaccines, oncolytic virotherapy, CAR-T cell therapy, and tumor-associated macrophage modulation, have shown promise. ICIs, successful in cancers like melanoma and non-small cell lung cancer, remain in early-stage research for GBM due to the CNS’s unique anatomy and immune microenvironment (13–17).

## 2 Immunological characteristics of glioblastoma

### 2.1 Anatomical and immune landscape

The CNS has traditionally been considered immune-privileged due to the blood-brain barrier (BBB), lack of lymphatic drainage, and limited immune cell presence, which restrict immune functions (18). However, recent studies have challenged this view, showing that glioma progression and inflammation increase BBB permeability, allowing immune cells and biomolecules to access the CNS (19–22). This disruption facilitates cytokine accumulation, exacerbating the immunosuppressive tumor microenvironment and reducing immunotherapy efficacy (23). Additionally, the discovery of functional lymphatic vessels in dural sinuses and the APC-like function of microglia and astrocytes has highlighted CNS-peripheral immune interactions (24, 25). Tumor-specific lymphocytes infiltrate the CNS through the BBB and choroid plexus, exerting cytotoxic effects on gliomas (24, 26, 27). Advances in nano-molecules and immune-targeting drugs have improved BBB penetration, enhancing intracranial tumor therapies (28). These findings advance glioma immunotherapy strategies.

### 2.2 Immune evasion characteristics and mechanisms of GBM

**Figure 1:**
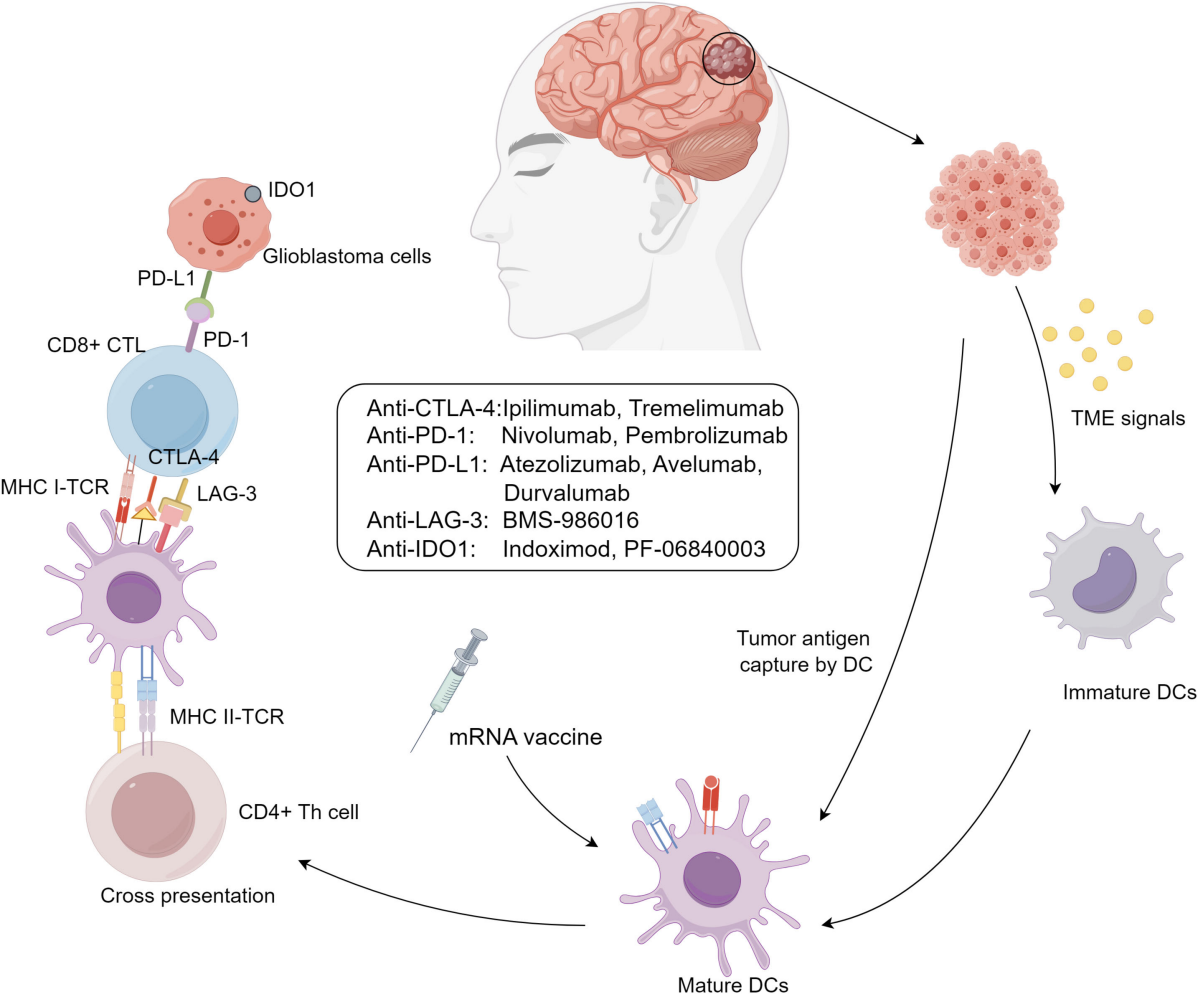
Immunotherapy approaches for glioblastoma.

GBM is characterized by its potent immune evasion capabilities, shaped by the brain’s unique immunological environment and a complex tumor microenvironment (TME). TME includes peripheral immune cells such as myeloid-derived suppressor cells (MDSCs) (29), natural killer (NK) cells, macrophages (30), neutrophils, CD4+ helper T cells (Th), CD8+ cytotoxic T lymphocytes (CTLs) (31), and regulatory T cells (Tregs), which influence tumor progression, recurrence, and resistance by modulating inflammatory responses (32). These immune cells are controlled by complex signaling networks, transforming into collaborators of tumor immune evasion and impairing immune surveillance (**Figure 1**).

GBM immune evasion mechanisms include: (I) downregulation of MHC-I molecules on tumor and antigen-presenting cells, limiting immune recognition; (II) activation of immune checkpoints like CTLA-4, PD-1/PD-L1, and LAG-3, suppressing immune responses; (III) secretion of immunosuppressive biomolecules such as TGF-β, IL-10, prostaglandin E2, and VEGF, creating a suppressive cytokine milieu; and (IV) infiltration of immunosuppressive cells like Tregs, M2-polarized tumor-associated macrophages (TAMs), and MDSCs, enhancing immunosuppression (33). These mechanisms are interdependent, amplifying immunosuppression, such as Tregs secreting TGF-β to suppress dendritic cell maturation and expressing CTLA-4 to inhibit effector T cell activation (34, 35). T-cell exhaustion also plays a critical role in GBM progression. This phenomenon, marked by the loss of effector functions and sustained expression of inhibitory receptors (e.g., PD-1, TIM-3, LAG-3), is induced by chronic antigen exposure and the immunosuppressive TME (36). Engagement of PD-1 with PD-L1 on tumor cells inhibits T-cell receptor (TCR) signaling, reducing cytokine production and cytotoxicity. Similarly, interactions between TIM-3 and galectin-9, and LAG-3 with MHC class II molecules, further suppress T-cell activity and contribute to exhaustion. The simultaneous expression of multiple immune checkpoints creates an environment that promotes tumor immune evasion and supports GBM growth, limiting the effectiveness of immunotherapies and contributing to GBM’s resilience against immune clearance.

Exogenous factors such as prolonged use of cytotoxic chemotherapy and immunosuppressive drugs, particularly glucocorticoids, exacerbate immune deficiencies in GBM patients, especially the elderly, leading to passive systemic immune suppression (37). However, studies in mouse models suggest that the CNS’s unique immune features may alleviate some glucocorticoid-induced effects on local tumor immunotherapy, offering insights into optimizing treatments, though human validation is needed (38). These immunological insights highlight the potential for targeted immunotherapies to reverse immunosuppression and induce durable anti-tumor immune responses, offering promising avenues for improving GBM treatment outcomes.

## 3 Advancements of IC inhibitors

The human immune system maintains equilibrium by activating immune cells through antigen and co-stimulatory signals while terminating responses via co-inhibitory receptors to prevent tissue damage. Tumor cells exploit these immune checkpoints (ICs) to evade surveillance, a key mechanism of immune escape (39). IC inhibitors, targeting these pathways, modulate immune cells in the tumor microenvironment rather than acting directly on tumor cells, making them critical tools in cancer immunotherapy with broad clinical applications (40).

### 3.1 Cytotoxic T-lymphocyte associated protein 4

CTLA-4 is expressed on activated T cells and Tregs that suppresses T cell activation by binding to CD80/CD86 and enhances Treg activity in the tumor microenvironment. Preclinical glioma studies show that CTLA-4 inhibitors promote CD4^+^ T cell proliferation and reduce Treg/CD4^+^ T cell ratios, correlating CTLA-4 expression with poor prognosis in glioblastoma (41, 42). Approved by the FDA for melanoma, ipilimumab and tremelimumab are under investigation for GBM. A Phase III trial (NCT02017717) comparing Ipilimumab and Nivolumab to bevacizumab reported partial responses and disease stabilization but was suspended due to subsequent failures (43, 44). Retrospective data links low CTLA-4 expression to improved survival (45). Interestingly, CTLA-4 blockade loses efficacy in CD4^+^ T cell-depleted mice, suggesting its anti-tumor effects rely on CD4^+^ T cell-mediated modulation of dendritic cells and microglia, offering new insights into its role in GBM treatment (46).

### 3.2 PD-1/PD-L1 pathway

Programmed cell death protein 1 (PD-1) and its ligands, PD-L1/PD-L2, suppress T cell activation and cytokine production, marking a key mechanism of immune evasion. PD-1 expression in GBM correlates with T cell exhaustion, reduced IFN-γ production, and poor survival (47–50). Tumor PD-L1 expression inhibits T cell activity and promotes resistance to cytotoxicity, accelerating glioblastoma progression (51, 52). Wild-type IDH glioblastomas exhibit higher PD-L1 levels than mutant forms, further linking the pathway to tumor grade and immune modulation (53, 54).

FDA-approved PD-1 inhibitors, such as Nivolumab and Pembrolizumab, have demonstrated efficacy in various cancers. In GBM, neoadjuvant PD-1 blockade enhances survival and local immune responses, though the microenvironment remains dominated by immunosuppressive myeloid cells (55, 56). Among 61 clinical trials, a completed study (NCT02337491) combining Pembrolizumab and Bevacizumab improved progression-free survival but increased toxicity (**Table 1**). Another (NCT02550249) revealed modest survival benefits with neoadjuvant Nivolumab but significantly boosted immune responses (57, 58). Trials on PD-L1 inhibitors like Avelumab, Durvalumab, and Atezolizumab continue, with most results pending, emphasizing the need for further optimization in this domain.

**Table 1:** Completed clinical trials evaluating immune checkpoint inhibitors in glioblastoma.

Target	Register number	Intervention	Disease	Phase	Sample size	Year (start/end)	mPFS (months)	mOS (months)	ORR
	NCT02550249	Nivolumab	GBM	II	29	2015/2017	4.1	7.3	60%
PD-1	NCT02337491	Pembrolizumab+Bevcizumab	GBM	II	50	2015/2018	4.1	8.8	20%
	NCT02337491	Pembrolizumab	GBM	II	30	2015/2018	1.4	10.3	0%
	NCT02968940	Avelumab	GBM	II	6	2017/2019	4.2	10.1	–

GBM, GlioblasIoma; mPFs, Median progression-free survival; mOS, Median overall survival; ORR, Overall radiographic response.

### 3.3 TIGIT/CD96

TIGIT and CD96 are critical co-inhibitory receptors in tumor immune regulation. CD96, part of the immunoglobulin superfamily, is a promising immunotherapy target due to its regulatory roles in NK and CD8+ T cell activity and influence on NK cell adhesion and migration. CD96 mitigates immune reactivation after PD-1/PD-L1 blockade, with elevated expression correlating with aggressive molecular phenotypes like IDH wild-type and mesenchymal subtypes. In GBM, CD96 co-expressed with PD-1 on CD8+ T cells indirectly promote tumor growth via enhanced IFN-γ secretion. Combined targeting of CD96, PD-1, and TIGIT has shown significant antitumor efficacy (59). Bioinformatics analyses reveal high CD96 mRNA expression correlates with invasiveness and poor prognosis in IDH wild-type GBM, underscoring its role as an independent prognostic factor (60). Similarly, TIGIT facilitates immune evasion by competing with CD115 for CD226 binding, while its ligand, CD155, overexpressed in GBM, enhances tumor migration and invasion. Monotherapy targeting TIGIT shows limited efficacy, but combining anti-TIGIT with anti-PD-1 significantly improves survival and immune response in GBM models (61). The TIGIT/CD155 axis also correlates with lower survival in lower-grade gliomas, making it a promising therapeutic target for GBM (61).

### 3.4 T cell immunoglobulin and mucin domain 3

TIM-3, an inhibitory checkpoint protein, marks T cell dysfunction in cancer and is highly expressed in tumor-infiltrating lymphocytes. It binds ligands such as galectin-9 (Gal-9), HMGB1, and CEACAM1, with the TIM-3/Gal-9 interaction suppressing Th1 responses and promoting Treg development. In GBM and IDH wild-type gliomas, TIM-3 is highly expressed and correlates with poor prognosis (62). TIM-3 knockout enhances NK cell cytotoxicity against GBM, supporting dual checkpoint blockade strategies for therapy (63). TIM-3 also contributes to chemoresistance in high-grade GBM, as silencing TIM-3 expression sensitizes tumor cells to temozolomide while inducing apoptosis (64). Dual TIM-3 and PD-1 blockade restores T cell function and demonstrates superior antitumor efficacy in preclinical models, with several anti-TIM-3 antibodies currently in clinical trials (65). These findings highlight TIM-3 as a critical target for immunotherapy in high-grade GBM.

### 3.5 Lymphocyte activation gene 3

Lymphocyte activation gene-3 (LAG-3) is a type I transmembrane protein structurally similar to CD4 and functions as an inhibitory co-receptor critical in autoimmune diseases, tumor immunity, and anti-infective immunity. As a second-generation immune checkpoint inhibitor target, LAG-3 represents a promising therapeutic direction after PD-1. It is minimally expressed on resting T cells but significantly upregulated on CD4 and CD8 T cells upon antigen stimulation and is closely tied to Treg homeostasis and function. LAG-3 often co-expresses with PD-1 on exhausted T cells, making it a key target in immunotherapy, particularly in combination with PD-1/PD-L1 inhibitors. As an early marker of T-cell exhaustion, LAG-3 inhibits T-cell activation by disrupting CD4-MHC II interactions and intracellular signaling (66) and directly suppresses CD8 T-cell functions.

In GBM, LAG-3 expression is associated with pathological subtypes, predominantly observed in high-grade GBM but absent in WHO grade II-III GBM. Preclinical studies showed that both LAG-3 inhibitor monotherapy and combination therapy with PD-1 inhibitors significantly prolonged survival in GBM mouse models. In human GBM, LAG-3 is mainly found on tumor-infiltrating lymphocytes (TILs) and perivascular lymphocytes (67). Despite promising preclinical data, the therapeutic implications in GBM remain uncertain, with clinical trials on the LAG-3 inhibitor BMS-986016 ongoing but unpublished.

### 3.6 IDO-targeted glioblastoma therapy

Indoleamine 2,3-dioxygenase (IDO), a tryptophan-metabolizing enzyme, plays a key role in glioma immune evasion by depleting tryptophan, essential for T-cell function, and promoting Treg infiltration (68, 69), This dual mechanism suppresses effector T-cell activity and facilitates glioma progression, with studies linking higher IDO expression to increased tumor malignancy and worse prognosis (69, 70). IDO inhibitors, including indoximod and PF-06840003, are under clinical investigation, with three trials currently registered. While one trial is completed, results are pending. Preliminary data suggest potential for IDO-targeted glioblastoma therapies, but further studies are needed to confirm their clinical efficacy and mechanisms.

## 4 Application of IC inhibitors in glioblastoma

While monotherapy with immune checkpoint inhibitors (ICIs) demonstrates limited efficacy in gliomas, combination strategies show significant promise. Ferroptosis, for example, plays a crucial role in reshaping the immunosuppressive microenvironment. The combination of ferrostatin-1 and anti-PD-L1 antibodies significantly prolonged survival, reduced tumor volume, and enhanced T-cell-mediated antitumor activity in GBM mouse models (71). Similarly, targeting PD-L1 expression, linked to glioblastoma cellular metabolism, with hexokinase inhibition and anti-PD-1 therapy significantly reduced immune evasion (72). Neutralizing IL-8 has been proposed as an adjunct to anti-PD-L1 therapy, offering further therapeutic potential (73). Approved ICIs include two anti-PD-1 antibodies (nivolumab and pembrolizumab) and three anti-PD-L1 antibodies (atezolizumab, avelumab, durvalumab) for solid tumors. Among glioblastoma patients, nivolumab has shown survival benefits in primary cases but limited efficacy in recurrent gliomas (58).

Dual immune checkpoint blockade holds potential for glioma therapy optimization. The FDA-approved combination of nivolumab and ipilimumab for hepatocellular carcinoma has shown promising preclinical results in glioma models, where CTLA-4 and PD-1 blockade significantly extended survival, with 74% of mice achieving long-term responses (74, 75). Combining CTLA-4 blockade with IL-12 enhanced TH1 polarization, achieving complete remission in glioma-bearing mice (76). Despite some durable responses, clinical efficacy remains constrained by immune-related adverse events, affecting 90% of patients and causing high-grade toxicities, particularly with high-dose ipilimumab. Only 20% of patients achieved stable disease at 12 weeks, with most discontinuing treatment due to progression or toxicity. A median follow-up revealed over half succumbed to the disease within 27.5 months (77), emphasizing the need for refined patient selection to improve outcomes and minimize adverse effects.

### 4.1 IC inhibitors combination

IC molecules, including PD-1, TIM-3, and LAG-3, regulate immune responses and are often co-expressed in various tumors, contributing to T-cell exhaustion and impaired function. Studies show that dual blockade of IC molecules restores T-cell function more effectively than single-agent inhibition, underscoring the synergistic potential of combination therapies (67, 78–81). For example, PD-1 and IDO induce CTLA-4 expression in Tregs, while CTLA-4 binding to CD80/CD86 upregulates IDO, highlighting interactions between pathways (82–84). Clinical trials increasingly adopt multi-ICI combinations to counteract T-cell dysfunction, though their efficacy in gliomas awaits further validation (82–84).

### 4.2 Combination of IC inhibitors and conventional therapy

Conventional cancer treatments—surgical resection, chemotherapy, and radiotherapy—often leave residual tumor cells that can lead to relapse and metastasis due to their resistance and extensive infiltration into surrounding tissues. To address this, recent strategies integrate immune checkpoint (IC) inhibitors with these traditional modalities, enhancing therapeutic efficacy through synergistic mechanisms. For instance, in brainstem glioma patients, the combination of the PD-1 inhibitor nivolumab with repeated radiotherapy has significantly improved overall survival, likely due to radiation-induced tumor antigen release that promotes immune cell infiltration into the tumor microenvironment (85, 86). Similarly, in murine glioma models, the co-administration of temozolomide with PD-1 or IDO inhibitors has markedly extended survival and inhibited tumor growth compared to monotherapy (87, 88). Despite these promising outcomes, further research is necessary to determine optimal dosing schedules and to evaluate potential additive toxicities when integrating IC inhibitors with conventional treatments.

### 4.3 Combination of IC inhibitors and anti-angiogenic therapy

Additionally, combining IC inhibitors with anti-angiogenic therapies represents a pivotal advancement in cancer treatment. Tumor-induced aberrant angiogenesis, primarily driven by pro-angiogenic factors like VEGF, creates a hypoxic microenvironment that suppresses anti-tumor immune responses and facilitates pro-tumor inflammatory cell infiltration, thereby limiting the efficacy of IC inhibitors (89). Preclinical studies have demonstrated that VEGF-blocking antibodies can downregulate multiple IC molecules, revealing the interplay between angiogenesis and immune suppression. The development of anti-angiogenic agents such as recombinant human endostatin has shown enhanced outcomes when used alongside PD-1 inhibitors in tumor models (90–92). Clinical trials are currently exploring combinations of IC inhibitors with anti-angiogenic drugs like bevacizumab, adatinib, axitinib, and endostar, which have shown preliminary efficacy in various solid tumors, including renal cell carcinoma, breast cancer, non-small cell lung cancer, and hepatocellular carcinoma. However, their effectiveness in glioma patients remains to be fully elucidated, necessitating further investigation to optimize these combination therapies for maximal clinical benefit.

### 4.4 Integration into novel treatment and comparison with standard therapies

Advancements in ICB therapy now encompass innovative approaches beyond traditional blocking antibodies. MicroRNAs (miRNAs) (93), such as miR-138 and miR-34a, have emerged as promising modulators of IC expression (94, 95). For instance, miR-138 suppresses CTLA-4 and PD-1 in T cells, while miR-34a downregulates PD-L1, enhancing anti-tumor immunity (96, 97). Additionally, targeting upstream regulators like PTEN and CDK5, which influence IC expression, can potentiate the efficacy of IC inhibitors (98, 99). Integrating these strategies with conventional treatments, such as chemotherapy and radiotherapy, may overcome resistance mechanisms and improve therapeutic outcomes. For example, combining miR-34a mimics with temozolomide has demonstrated synergistic effects in preclinical models, resulting in increased apoptosis and reduced tumor growth.

Compared to traditional therapies, these emerging strategies offer enhanced specificity and the ability to target multiple oncogenic pathways simultaneously, reducing the likelihood of resistance and minimizing collateral damage to healthy tissues. Furthermore, personalized treatment regimens based on miRNA profiles and upstream regulator status can tailor therapies to individual tumor characteristics, improving efficacy and reducing adverse effects. This integration not only complements existing modalities but also addresses the molecular mechanisms underlying glioblastoma’s immune evasion, providing a more robust and sustained anti-tumor response (100).

## 5 Conclusion

Tumor cells enhance their proliferative, invasive, and metastatic capabilities through complex endogenous and environmental regulatory mechanisms, with immune evasion being a multifactorial process involving extensive regulatory networks. Monotherapies targeting single pathways, such as PD-1 inhibitors like Nivolumab or Pembrolizumab, have shown limited success in prolonging progression-free survival in recurrent high-grade gliomas. This underscores the adaptability and diversity of tumor immune evasion strategies, indicating that single immune checkpoint blockade is often insufficient.

Consequently, multi-target combination therapies have become a focal point in cancer immunotherapy. These multidimensional approaches integrate multiple immune regulatory mechanisms to effectively counteract the intricate immune evasion networks employed by tumors. Strategies such as combining immune checkpoint inhibitors with metabolic regulation targets, utilizing gene editing technologies to modulate immune cell functions, and applying single-cell multi-omics to analyze immune microenvironment changes post-treatment hold significant promise. These emerging combination therapies are poised to enhance therapeutic efficacy and offer breakthrough options for treating aggressive cancers, including high-grade gliomas.
